# Structural versatility of the quasi-aromatic Möbius type zinc(ii)-pseudohalide complexes – experimental and theoretical investigations†

**DOI:** 10.1039/c9ra05276c

**Published:** 2019-07-31

**Authors:** Mariusz P. Mitoraj, Farhad Akbari Afkhami, Ghodrat Mahmoudi, Ali Akbar Khandar, Atash V. Gurbanov, Fedor I. Zubkov, Rory Waterman, Maria G. Babashkina, Dariusz W. Szczepanik, Himanshu S. Jena, Damir A. Safin

**Affiliations:** Department of Theoretical Chemistry, Faculty of Chemistry, Jagiellonian University Gronostajowa 2 30-387 Cracow Poland mitoraj@chemia.uj.edu.pl; Department of Inorganic Chemistry, Faculty of Chemistry, University of Tabriz 51666-16471 Tabriz Iran; Department of Chemistry, Faculty of Science, University of Maragheh P.O. Box 55181-83111 Maragheh Iran mahmoudi_ghodrat@yahoo.co.uk; Department of Chemistry, Baku State University Z. Xalilov Str. 23 AZ1148 Baku Azerbaijan; Centro de Química Estrutural, Instituto Superior Técnico, Universidade de Lisboa Av. Rovisco Pais 1049-001 Lisboa Portugal; Organic Chemistry Department, Faculty of Science, Peoples' Friendship University of Russia (RUDN University) 6 Miklukho-Maklaya St. Moscow 117198 Russian Federation; Department of Chemistry, University of Vermont 82 University Place Burlington VT 05405 USA; Institute of Chemistry, University of Tyumen Perekopskaya Str. 15a 625003 Tyumen Russian Federation damir.a.safin@gmail.com d.a.safin@utmn.ru; COMOC, Department of Chemistry, Ghent University Krijgslaan 281-S3B Ghent-9000 Belgium

## Abstract

In this contribution we report for the first time fabrication, isolation, structural and theoretical characterization of the quasi-aromatic Möbius complexes [Zn(NCS)_2_L^I^] (1), [Zn_2_(μ_1,1_-N_3_)_2_(L^I^)_2_][ZnCl_3_(MeOH)]_2_·6MeOH (2) and [Zn(NCS)L^II^]_2_[Zn(NCS)_4_]·MeOH (3), constructed from 1,2-diphenyl-1,2-bis((phenyl(pyridin-2-yl)methylene)hydrazono)ethane (L^I^) or benzilbis(acetylpyridin-2-yl)methylidenehydrazone (L^II^), respectively, and ZnCl_2_ mixed with NH_4_NCS or NaN_3_. Structures 1–3 are dictated by both the bulkiness of the organic ligand and the nature of the inorganic counter ion. As evidenced from single crystal X-ray diffraction data species 1 has a neutral discrete heteroleptic mononuclear structure, whereas, complexes 2 and 3 exhibit a salt-like structure. Each structure contains a Zn^II^ atom chelated by one tetradentate twisted ligand L^I^ creating the unusual Möbius type topology. Theoretical investigations based on the EDDB method allowed us to determine that it constitutes the quasi-aromatic Möbius motif where a metal only induces the π-delocalization solely within the ligand part: 2.44|*e*| in 3, 3.14|*e*| in 2 and 3.44|*e*| in 1. It is found, that the degree of quasi-aromatic π-delocalization in the case of zinc species is significantly weaker (by ∼50%) than the corresponding estimations for cadmium systems – it is associated with the Zn–N bonds being more polar than the related Cd–N connections. The ETS-NOCV showed, that the monomers in 1 are bonded primarily through London dispersion forces, whereas long-range electrostatic stabilization is crucial in 2 and 3. A number of non-covalent interactions are additionally identified in the lattices of 1–3.

## Abstract 2

Interplay between various types of non-covalent interactions allowed for isolation of rare examples of Zn(ii) based quasi-aromatic Möbius type species.

## Graphical abstract

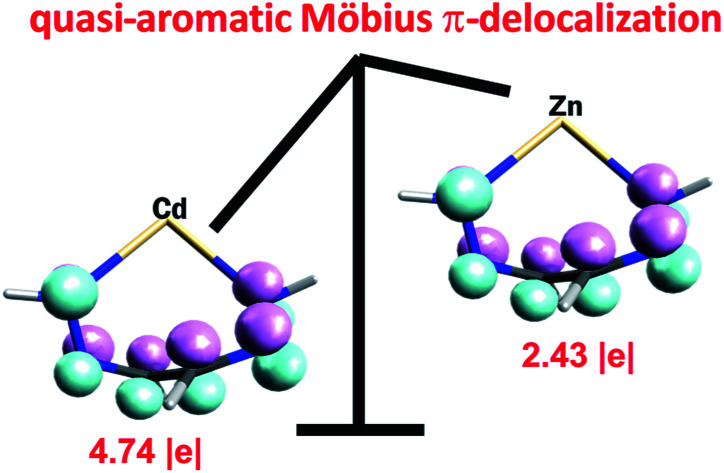

## Introduction

Helical molecules are highly favoured by nature.^1^ Such molecules are of great importance, which is also supported by the structure of deoxyribonucleic acid first discovered in 1953.^2^

On the other hand, zinc(ii) (Zn^II^) ions are found in all six main classes of metalloenzymes and are essential for living organisms.^3,4^ Moreover, the dinuclear Zn^II^ complex fabricated from doubly deprotonated octaethyl formylbiliverdine is the first established helical doublestranded structure, which was reported in 1976.^5^ Following this discovery, strategies towards helical structure as well as their self-assembly have been the focus of researchers.^6,7^ Obviously, the most powerful strategy towards metal-based helical structures is the smart predesign of parent ligands. The other strategy, which, however, is less predictable and thus less efficient, is the choice of a metal-containing precursor. The latter is much less investigated.^5–7^

Some efforts have been focused on the design and preparation of helical metal complexes by applying chelating ligands with suitable donor sites.^7–16^ The point is that the ligand should produce a helical topology upon binding to the metal ions. In some cases, the coordination features of the cations dictate the wrapping of non-helical chelating ligands around them in such a manner that they can be twisted and eventually form helical complexes.^7,9–11,13,15^ However, synthesis of organic ligands with a helical topology is more difficult than metallo-organic compounds and there are only a few reported synthetic helical organic molecules.^8,12,14,16,17^ Researchers mainly focused on the design and construction of metal complexes with synthetic helical chelating ligands.^18–20^ Recently, we have also directed our attention to Schiff bases comprising two pyridyl-imine functions obtained from benzyldihydrazone.^21–27^ These ligands were found to be efficient for helical structures upon coordination to metal centers. Particularly our comprehensive efforts were directed to various Cd^II^ salts as complexing agents.^23–27^ Moreover, we were able to demonstrate for the first time that the helical motif in the obtained complexes together with the chelate metalloring correspond to a quasi-aromatic Möbius object.^24,27^

Herein, we report Zn(NCS)_2_- and Zn(N_3_)_2_-derived structures with 1,2-diphenyl-1,2-bis((phenyl(pyridin-2-yl)methylene)hydrazono)ethane (L^I^) and benzilbis(acetylpyridin-2-yl)methylidenehydrazone (L^II^). Using thiocyanate (NCS^−^) and azide (N_3_^−^) counterions is intriguing and of great interest since both anions are known to be ambidentate ligands, which can bind metal centers in different coordination modes.^28,29^ As a result were able to isolate the unique quasi-aromatic Möbius type zinc complexes [Zn(NCS)_2_L^I^] (1), [Zn_2_(μ_1,1_-N_3_)_2_(L^I^)_2_][ZnCl_3_(MeOH)]_2_·6MeOH (2) and [Zn(NCS)L^II^]_2_[Zn(NCS)_4_]·MeOH (3), respectively. Notably, our numerous attempts to isolate crystals of the reaction product of Zn(N_3_)_2_ and L^I^ failed regardless using a great number of Zn^II^ and N_3_^−^ sources. DFT experiments were additionally performed to estimate the stability and aromaticity of the obtained species.

## Results and discussion

**Scheme 1:**
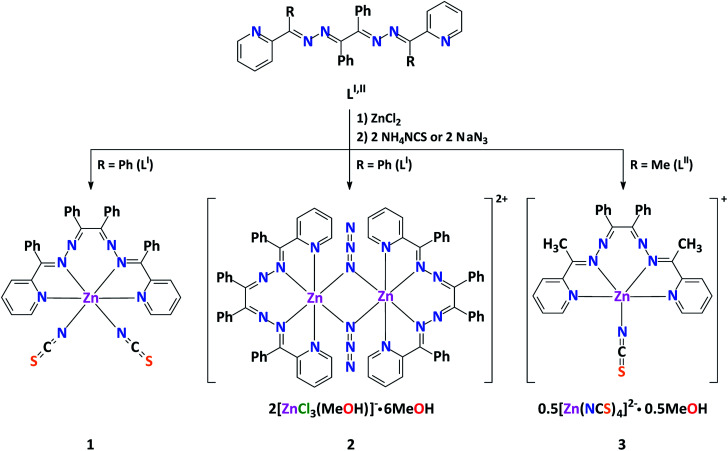
Synthesis of complexes 1–3.

Interaction of ZnCl_2_ mixed with NH_4_NCS or NaN_3_ with L^I^ or L^II^ in MeOH at 60 °C has allowed to isolate complexes 1–3 (Scheme 1 and ESI†). The elemental analysis data supports their compositions. Notably, the same one-pot reaction of L^I^ or L^II^ with Cd(NO_3_)_2_ in the presence of NH_4_NCS produced a dinuclear structure [Cd_2_(μ_1,3_-NCS)_2_(NCS)_2_(L^III^)_2_]·4MeOH (4), where L^III^ is formed upon hydrolysis of one of the 2-PyC(Ph) functions of L^I^,^27^ and neutral mononuclear complex [Cd(NCS)_2_(L^II^)(MeOH)] (5),^24^ respectively.

The FTIR spectrum of 1 contains a characteristic intense band at 2073 cm^−1^ attributed to the CN stretching of NCS^−^. The same stretching mode in the IR spectrum of 3 is shown as two clearly defined bands at 2066 and 2112 cm^−1^, corresponding to two different types of the NCS^−^ ligands (Scheme 1). These bands are in the typical region for the N-linked terminal NCS^−^ ions.^30^ The N_3_^−^ anions in the FTIR spectrum of 2 are shown as an intense band at 2054 cm^−1^ arising from the *ν*_asym_ stretching vibration, as well as a band at 1441 cm^−1^ corresponding to the *ν*_asym_ stretching vibration. The C=N stretching vibration is at a lower energy by 40 cm^−1^ for complexes 1 and 2 compared to the free ligand L^I^, whilst a similar 20 cm^−1^ difference is observed in this vibration between compound 3 and L^II^.^22^ This firmly confirms the participation of the azomethine nitrogen atoms in chelate formation. The FTIR spectra of 2 and 3 further contain a broad band for the methanol at 3353 to 3443 cm^−1^, respectively.

Complex 1 crystallizes in the monoclinic space group *P*2_1_/*n*, while complexes 2 and 3 each crystallize in the triclinic space group *P*1̄. It is worthy to note, that 1 is isostructural to its Mn^II^ and Co^II^ analogues.^22,31^

**Fig. 1:**
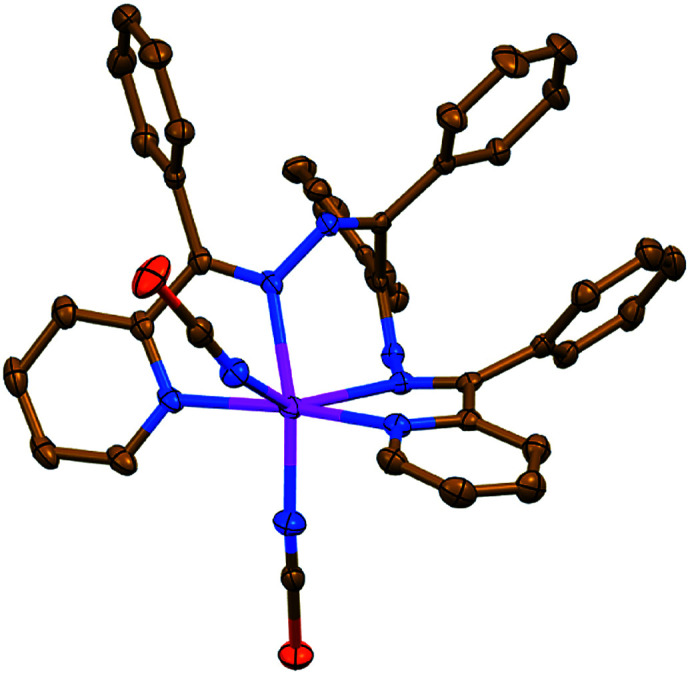
Crystal structure of 1 (H-hydrogen atoms are omitted for clarity). Color code: C = gold, N = blue, S = orange, Zn = magenta.

Complex 1 has a neutral discrete heteroleptic mononuclear structure, where the Zn^II^ metal center is coordinated by one ligand L^I^*via* its two pyridyl-imine chelate functions as well as two N-bound NCS^−^ anions giving rise to the ZnN_6_ chromophore with a distorted trigonal-prismatic coordination environment around the cation (Fig. 1, Table S1 in the ESI†), which has been proven by the SHAPE 2.1 software.^32,33^

**Fig. 2:**
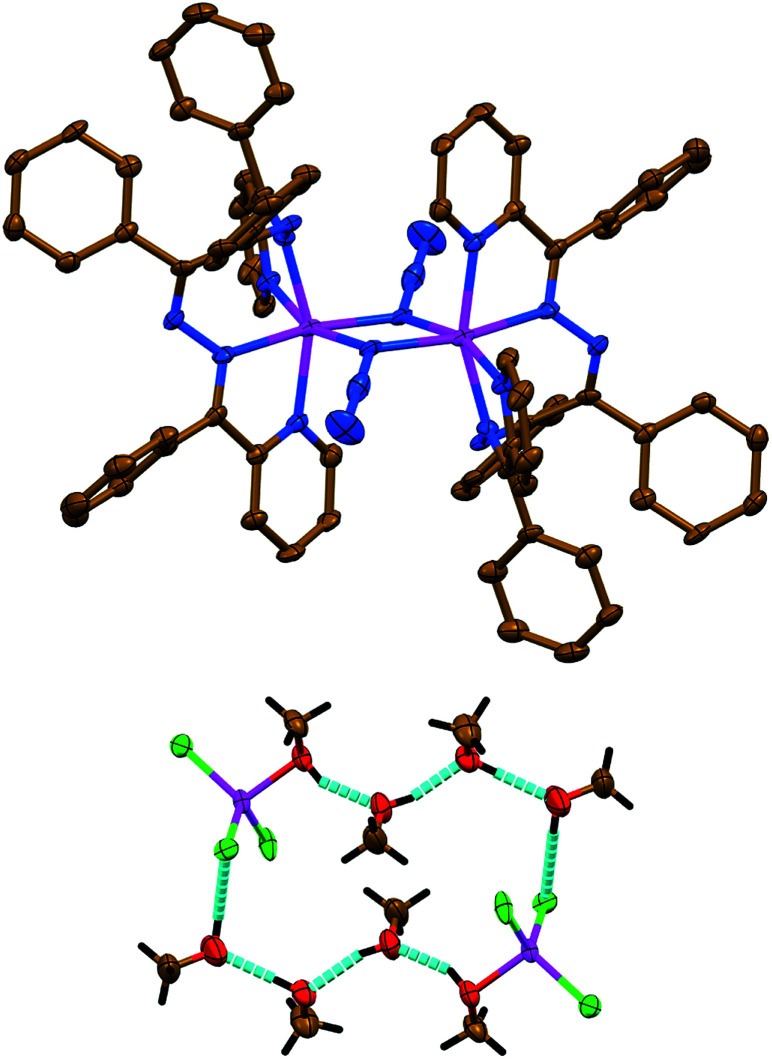
(top) Crystal structure of the cationic part [Zn_2_(μ_1,1_-N_3_)_2_(L^I^)_2_]^2+^ of 2. Hydrogen atoms are omitted for clarity. Color code: C = gold, N = blue, Zn = magenta. (bottom) Crystal structure of the hydrogen bonded synthon of motif *R*^8^_8_(20) of the ([ZnCl_3_(MeOH)]_2_)^2−^·6MeOH composition, constructed from the [ZnCl_3_(MeOH)]^−^ anionic part and lattice MeOH molecules of 2. Color code: H = black, C = gold, Cl = green, O = red, Zn = magenta.

Complexes 2 and 3 each exhibit a salt-like structure (Scheme 1), as opposed to our previously studied Cd^II^ based counterparts.^23–27^ In 2, the cationic part exhibits a doubly charged centrosymmetric dinuclear structure, were two Zn^II^ centers are interlinked *via* two μ_1,1_-N_3_^−^ anions and the coordination domain of each metal is filled by the tetracoordinated ligand L^I^ (Fig. 2). Here a coordination geometry is best described as a distorted octahedron (Table S1 in the ESI†).^32,33^ The anionic part represents a discrete mononuclear structure of the composition [ZnCl_3_(MeOH)]^−^ with a tetracoordinate Cl_3_O environment around the metal atom (Fig. 2). As evidenced from the so-called distortion index *τ*_4_ = 0.9513,^34^ the coordination core of [ZnCl_3_(MeOH)]^−^ is almost a perfect tetrahedron. This is also supported by the SHAPE 2.1 software (Table S1 in the ESI†).^32,35^

The anionic part of 2*via* one of its chlorine atoms and the methanol OH hydrogen atom is engaged in intermolecular hydrogen bonds with the lattice MeOH molecules yielding a synthon of motif *R*^8^_8_(20) of the ([ZnCl_3_(MeOH)]_2_)^2−^·6MeOH composition (Fig. 2, Table S2 in the ESI†).

**Fig. 3:**
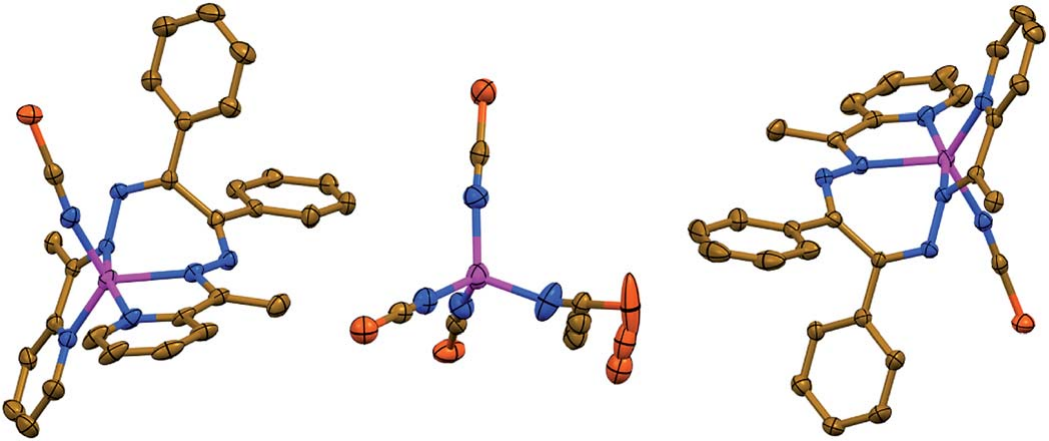
Crystal structure of 3 (hydrogen atoms and MeOH molecules are omitted for clarity). Color code: C = gold, N = blue, S = orange, Zn = magenta.

The salt like structure of 3 is built from two [Zn(NCS)L^II^]^+^ cations, where the Zn^II^ metal center is, similar to 1 and 2, chelated by two pyridyl-imine fragments of one parent ligand L^II^, and further bound by one N-linked NCS^−^ anion, exhibiting a pentacoordinated geometry (Fig. 3). The distortion index *τ*_5_^34^ is 0.693 and 0.653 for two [Zn(NCS)L^II^]^+^ cations. These values are best described as being about 31% and 35%, respectively, along the pathway of distortion from the ideal trigonal bipyramidal structure towards square pyramidal structure. The trigonal bipyramidal coordination environment is also evidenced from the SHAPE 2.1 software (Table S1 in the ESI†).^32,36^

The anionic part of 3 exhibits a doubly charged [Zn(NCS)_4_]^2−^ species, where four NCS^−^ anions are bound *via* their N-atoms yielding a tetracoordinated coordination geometry around the metal atom (Fig. 3). The *τ*_4_ value of 0.9594 indicates almost a perfect tetrahedron, which has also been supported by the SHAPE 2.1 software (Table S1 in the ESI†).^32,35^ Notably, the CS fragment of one of the NCS^−^ anions is disordered over three positions with a ratio of 35% : 35% : 30% (Fig. 3).

The Zn–N bonds in 1–3, formed by four nitrogen atoms of the corresponding organic ligands, are in the range from 2.073(3) Å to 2.2960(15) Å, maintaining that Zn–N_Py_ < Zn–N_imine_ (Table 1). It is worthy to note, that the Cd(L)–NCS bonds (1.970(3)–2.0289(18) Å) in 1 and 3 are remarkably shorter than the Zn–N_Py_ and Zn–N_imine_ bonds, while the Zn(L)–N_3_ bonds in 2 are similar to those within the corresponding organic ligand. This is obviously explained by the terminal coordination mode of the NCS^−^ anions in contrast to the μ_1,1_-coordination mode of N_3_^−^. The Zn–NCS bonds within the [Zn(NCS)_4_]^2−^ anion in 3 are 1.953(6)–1.983(4) Å. All the NCS^−^ and N_3_^−^ ligands are almost linear (Table 1). The Zn⋯Zn separation within the dinuclear molecule of 2 is 3.3729(15) Å.

**Table 1:** Selected bond lengths (Å) and angles (°) for 1–3

	Complex 1	Complex 2	Complex 3
**Bond lengths**			
Zn–N_Py_	2.2165(16), 2.2755(17)	2.075(5), 2.168(5)	2.073(3), 2.079(4), 2.100(3)
Zn–N_imine_	2.2692(16), 2.2960(15)	2.131(5), 2.265(5)	2.107(3), 2.118(3), 2.119(3), 2.123(4)
Zn(L)–NCS	2.0046(18), 2.0289(18)	—	1.970(3), 1.971(3)
Zn(L)–N_3_	—	2.154(5), 2.225(4)	—
Zn–NCS	—	—	1.953(6), 1.955(5), 1.971(5), 1.983(4)
Zn(L)⋯Zn(L)	—	3.3729(15) (intramolecular)	—

**Bond angles**			
N_Py_–Zn–N_Py_	170.55(6)	103.76(17)	104.50(14), 104.63(15)
N_Py_–Zn–N_imine_	69.96(6), 70.63(6), 116.80(6), 118.40(6)	75.27(17), 75.95(17), 102.79(17), 156.78(18)	76.44(12), 76.71(13), 78.20(14), 78.36(14), 119.93(12), 121.35(14), 163.40(13), 164.34(13)
N_imine_–Zn–N_imine_	76.13(6)	81.62(17)	86.81(12), 87.76(12)
N_Py_–Zn–NCS	86.22(7), 87.06(7), 87.39(6), 89.72(7)	—	97.89(14), 98.68(13), 122.76(15), 124.22(15)
N_imine_–Zn–NCS	88.08(6), 93.14(7), 142.43(6), 144.78(7)	—	94.12(13), 94.20(13), 114.07(15), 114.42(16)
N_Py_–Zn–N_3_	—	89.25(17), 92.07(17), 98.01(18), 161.65(17)	—
N_imine_–Zn–N_3_	—	89.89(17), 93.84(17), 105.15(17), 167.47(17)	—
NCS–Zn(L)–NCS	117.54(7)	—	—
NCS–Zn–NCS	—	—	105.0(2), 105.3(2), 110.0(2), 112.00(17), 112.0(2), 112.73(17)
N_3_–Zn–N_3_	—	79.26(17)	—
Zn(L)–N–C(S)	158.37(16), 156.48(18)	—	165.7(4), 166.8(4)
Zn–N–C(S)	—	—	149.5(11), 150.9(15), 158(3), 163.5(5), 169.6(4), 174.5(4)
Zn–N–N(N)	—	121.8(4), 123.4(4)	—
N–C–S	178.62(19), 178.9(2)	—	178.6(4), 179.2(4)
N–N–N	—	178.8(7)	—
Zn–N–Zn	—	100.7(2)	—

**Torsion angles** a			
N–C(Ph)–C(Ph)–N	−67.4(3)	−64.7(9)	66.1(6), 67.6(6)
C(Ph)–N–N–C(Ph)	−87.0(2), −114.85(19)	−96.7(7), −143.5(6)	—
C(Me)–N–N–C(Ph)	—	—	138.5(4), 140.0(4), 152.4(4), 152.9(4)
Py⋯Py	64.19(10)	51.1(3)	56.6(2), 58.0(2)
Zn–N–N–C(Ph)	81.04(18), 86.13(17)	49.8(7), 81.7(5)	−47.0(5), −48.3(5), −71.2(3), −72.4(4)

a Torsion angles must be compared by their magnitudes.

Organic ligands in the structures of 1–3 each produce a twisted geometry of different extent. As a result of this conformation, the N–C(Ph)–C(Ph)–N fragments adopt a torsion angle of about 65° (Table 1), which is significantly lower than in the corresponding Cd^II^ analogues.^23–27^ This is also reflected in the Zn–N–N–C(Ph), C(Ph)–N–N–C(Ph) in 1 and 2, and C(Me)–N–N–C(Ph) in 3 torsion angles. Particularly, a simultaneous influence of a pentacoordination mode of the metal center together with the presence of less bulkier Me substituents in complex 3 leads to the C(Me)–N–N–C(Ph) torsion angles of close values (∼138–153°), while a hexacoordination mode of Zn^II^ and the presence of the organic ligand, which is highly enriched by four phenyl fragments, induces significantly different C(Ph)–N–N–C(Ph) torsion angles within single species (Table 1). The Zn–N–N–C(Ph) torsion angles are very similar in 1, while the same angles in 2 and 3 are significantly different (Table 1). The torsion angle between two pyridyl rings ranges from about 51° to 64°, increasing from 2 through 3 to 1 (Table 1).

Due to a twisted helical topology of organic ligands in the mononuclear structures of 1 and 3, enantiomers of the coordination species can be expected. Indeed, both structures exhibit molecules with Δ and Λ helicity. The overall structure of 1 and 3 is a racemic mixture. The molecule of 2, although also containing organic ligands with a twisted helical topology, is constructed from two chiral centres, resulting in the formation of the achiral meso-form.

The crystal packing of 1–3 is described by a network of face-to-face π⋯π stacking between the aromatic rings (Table S3 in the ESI†). The structures of 1 and 2 are also dictated by C–H⋯π interactions (Table S4 in the ESI†).

For more detailed analyses of non-covalent interactions in 1–3 the charge and energy decomposition scheme ETS-NOCV^37^ is applied as available in the ADF program.^38^ We have applied BLYP-D3/TZP since they provide reliable results for non-covalent interactions.^39^ The X-ray models are considered.

**Fig. 4:**
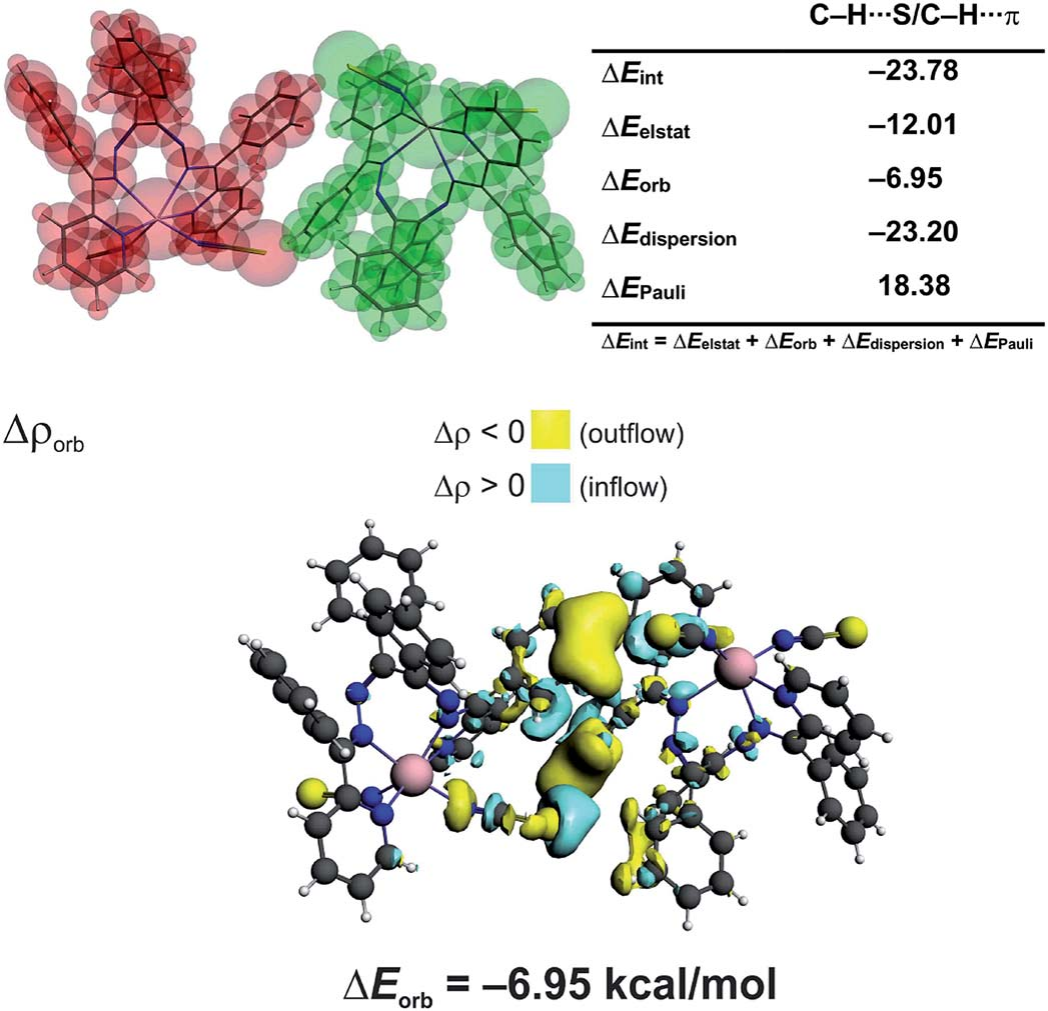
(top) ETS-NOCV outcomes scrutinizing the nature of bonding between the [Zn(NCS)_2_L^I^] monomers in 1. (bottom) The overall change in electron density Δ*ρ*_orb_ with the corresponding energy Δ*E*_orb_.

We have determined, that the neutral monomers of [Zn(NCS)_2_L^I^] in 1 are efficiently bonded to each other with the interaction energy Δ*E*_int_ = −23.78 kcal mol^−1^ (Fig. 4). The main gluing force (55% of the overall stabilization) is the dispersion term (Δ*E*_disp_ = −23.20 kcal mol^−1^) due to the presence of C–H⋯S and C–H⋯π contacts (Fig. 4). Such close contacts enforce additionally less important electrostatic (28.5%, Δ*E*_elstat_ = −12.01 kcal mol^−1^) and charge delocalisation (16.5%, Δ*E*_orb_ = −6.95 kcal mol^−1^) constituents (Fig. 4). The prevalence of the Δ*E*_disp_ term is consistent with recent findings, which rediscover the importance of London dispersion forces in small and sizeable species.^24,27,40–56^

**Fig. 5:**
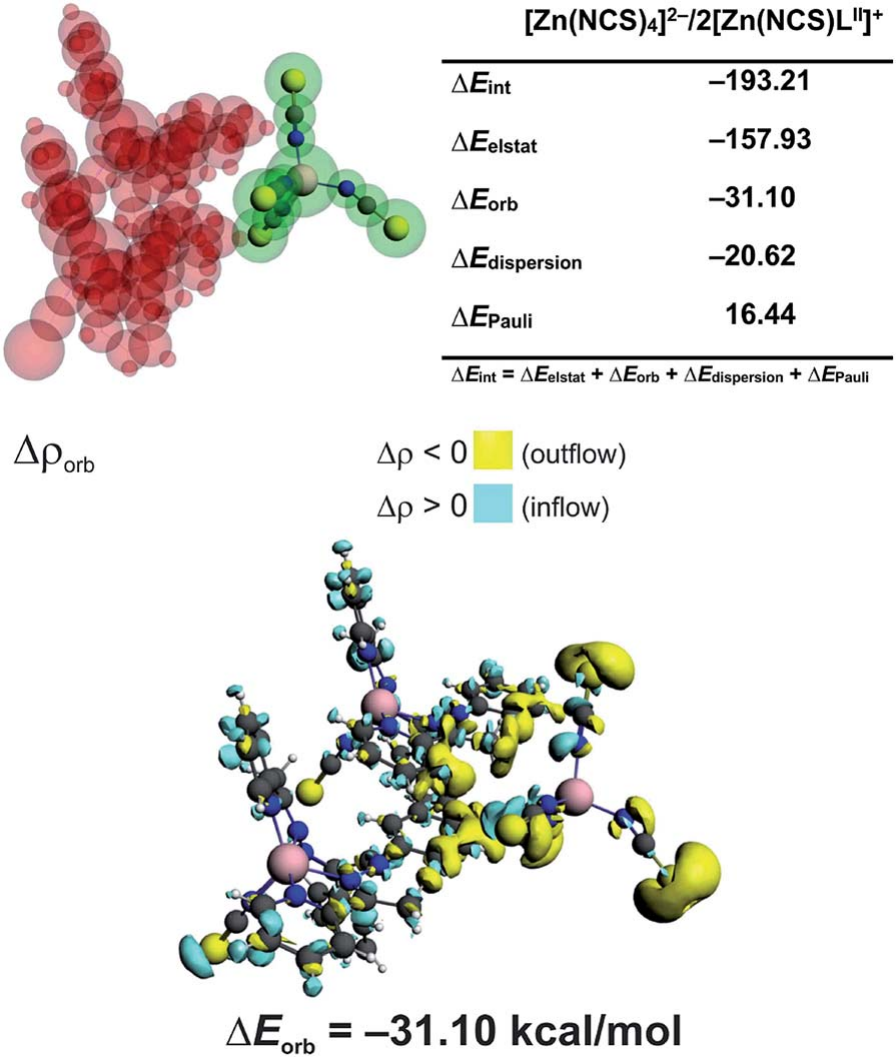
(top) ETS-NOCV outcomes scrutinizing the nature of ionic interaction between [Zn(NCS)_4_]^2−^ and two [Zn(NCS)L^II^]^+^ in 3. (bottom) The overall change in electron density Δ*ρ*_orb_ with the corresponding energy Δ*E*_orb_.

**Fig. 6:**
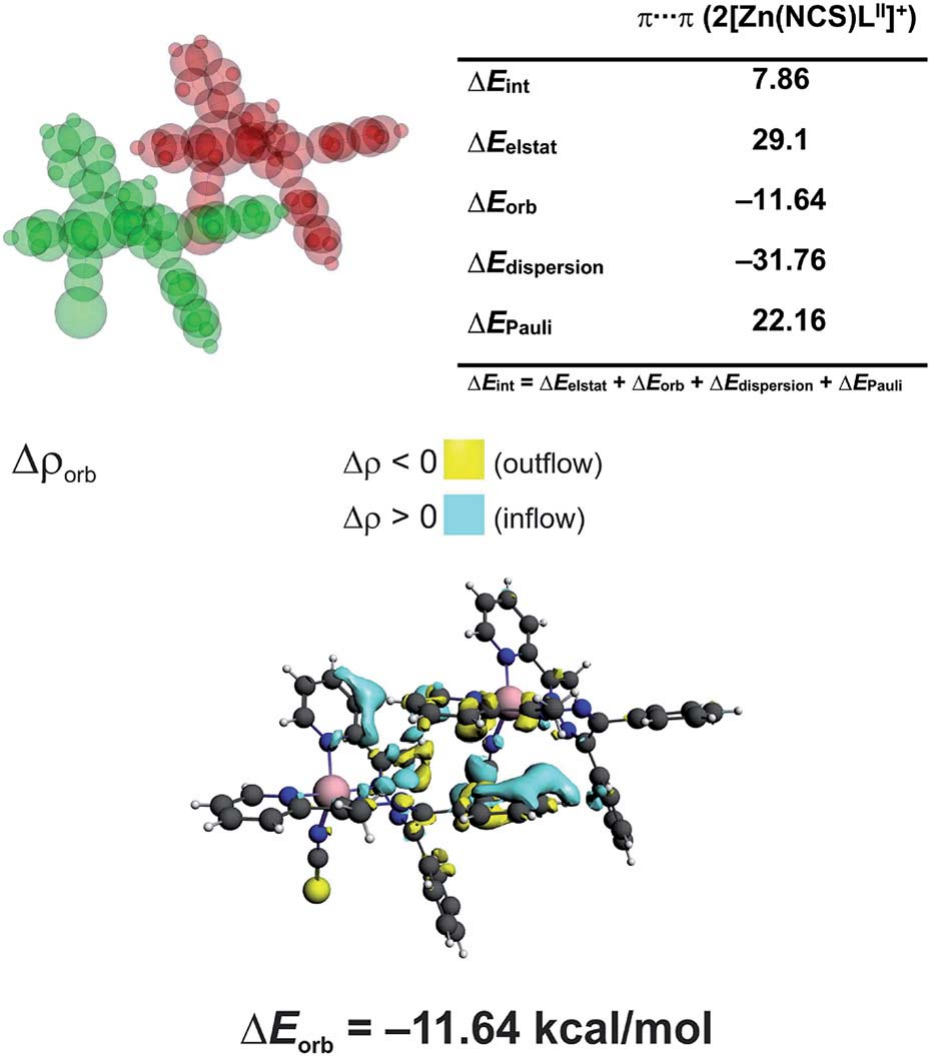
(top) ETS-NOCV outcomes scrutinizing the nature of ionic interaction between two π-stacked [Zn(NCS)L^II^]^+^ units in 3. (bottom) The overall change in electron density Δ*ρ*_orb_ with the corresponding energy Δ*E*_orb_.

In 3 the [Zn(NCS)_4_]^2−^ anion sticks very strongly (Δ*E*_int_ = −193.21 kcal mol^−1^) to two neighboring stacked [Zn(NCS)L^II^]^+^ units primarily through electrostatic forces (75.3% of the overall stabilization) (Fig. 5). Quite notable (14.8%) is the charge delocalization term Δ*E*_orb_ = −31.10 kcal mol^−1^ mostly due to C–H⋯π contacts followed by the least important (9.90%) dispersion term Δ*E*_disp_ = −20.62 kcal mol^−1^ (Fig. 5). It is interesting to emphasize, that such ionic bonds are of crucial importance for the overall stability of 3 since the pure π⋯π stacking between the [Zn(NCS)L^II^]^+^ units, though containing a significant portion of dispersion stabilization (Δ*E*_disp_ = −31.76 kcal mol^−1^), is found to be repulsive with Δ*E*_int_ = 7.86 kcal mol^−1^ caused predominantly by the unfavourable electrostatic constituent Δ*E*_elstat_ = 29.1 kcal mol^−1^ (Fig. 6). In 2 the electrostatically dominated stabilizing interactions occur between [ZnCl_3_(MeOH)]^−^ and [Zn_2_(μ_1,1_-N_3_)_2_(L^I^)_2_]^2+^ (Fig. S1 in the ESI†). Additionally, [ZnCl_3_(MeOH)]^−^ forms primarily O–H⋯O as well as a series of ancillary C–H⋯Cl hydrogen bonds with the neighbouring methanol species. Such cooperative interactions, leading to Δ*E*_int_ = −13.17 kcal mol^−1^, are found to be determined mostly by the electrostatic factor (50%) followed by the charge delocalization (27%) and dispersion (23%) constituents (Fig. S2 in the ESI†). The synthon [Zn_2_(μ_1,1_-N_3_)_2_(L^I^)_2_]^2+^ was found to be stable due to electrostatically dominated (Δ*E*_elstat_ = −63.33 kcal mol^−1^) dative-covalent Zn–N connections with Δ*E*_int_ = −31.63 kcal mol^−1^ constituted additionally from the significant portion of London dispersion forces (Δ*E*_disp_ = −37.25 kcal mol^−1^) (Fig. S3 in the ESI†).

In order to evaluate aromaticity in 1–3, we have applied the electron density of delocalized bonds (EDDB) method, which has been proposed to visualize and quantify aromaticity and chemical resonance in a wide range of chemical species.^57–60^

Moreover, it has recently been shown that, in the case of organometallics, the EDDB method provides very useful data on the role of the transition metal d-orbitals in electron delocalization,^24,27,50,61^ which is inaccessible by means of such popular and commonly used aromaticity descriptors as the nucleus-independent chemical shift (NICS)^62^ or the anisotropy of the induced current density (ACID).^63^

**Fig. 7:**
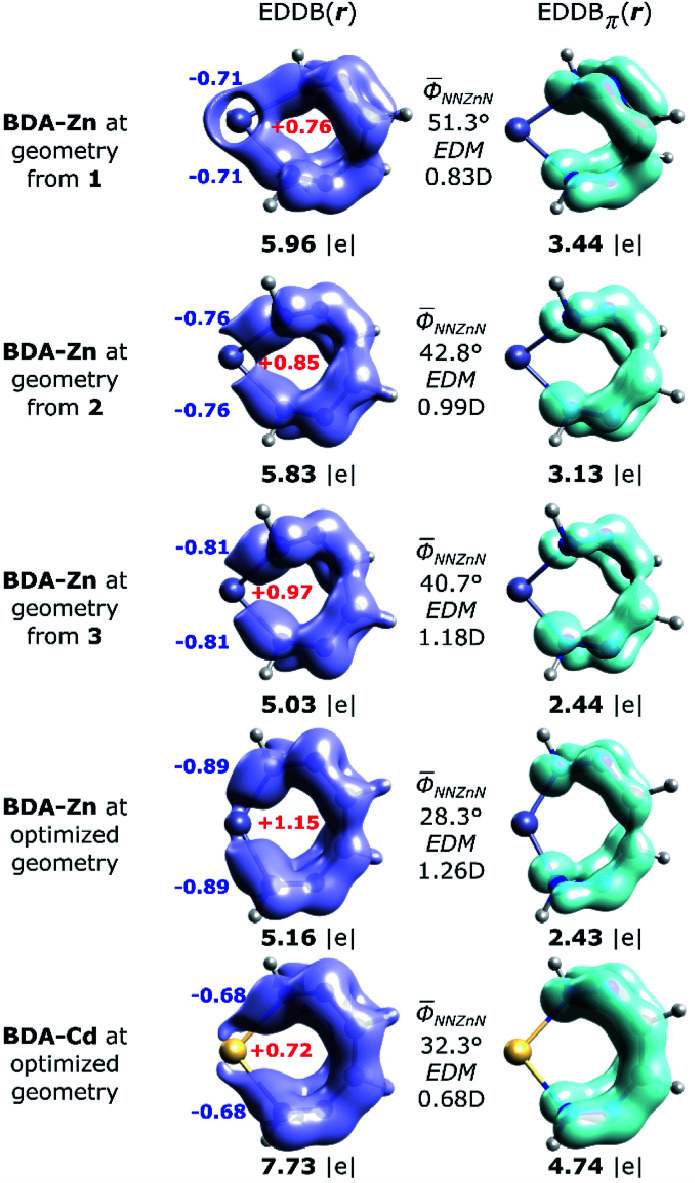
Imagining of the EDDB(r) and EDDB_π_(r) functions with the corresponding electron populations (in |*e*|) for the isolated 7-MR model systems at geometries adopted from the corresponding crystals of 1–3, as well as the fully optimized units: BDA–Zn and BDA–Cd.^27^ The natural atomic charges (colored bold numbers), average dihedral angles and the calculated electric dipole moments (EDM) have been added for comparison.

The global EDDB isocontours and the corresponding electron populations of 1–3 are collected in Fig. S4 in the ESI.† Here, we focus our attention on the characteristic seven-membered quasi-aromatic motif (7-MR), encompassing the twisted 1,1′-(1,2-ethenediyl)bis-diazene (BDA) fragment and the metal atom (abbreviated as BDA–Zn). The BDA-based complexes with cadmium have recently been demonstrated to exhibit a unique type of transition-metal induced Möbius-like aromaticity in which the metal d-orbitals themselves do not contribute to the π-conjugation occurring at BDA.^24,27^ Since the quantitative study of aromaticity/electron delocalization in large systems is very difficult in practice, we have decided to consider the simplified BDA–Zn models adopting the exact fragments geometries from crystals of 1–3 (Fig. 7). The calculated total EDDB contours and electron populations have been dissected (according to the orbital symmetry) to get the strict π-contributions to quasi-aromaticity; natural atomic charges on the metal and the two closest nitrogen atoms have been added together with the average dihedral angles and the calculated electric dipole moments (EDM). It was found, that the number of π-electrons delocalized in the quasi-aromatic rings, particularly in 1 and 2 (on average ∼3.3|*e*|), resembles pretty much the values found for the previously studied BDA–Cd complexes, despite different configurations of the phenyl units and applying other ligands.^24,27^ The most twisted 7-MR in 1 (containing the most bulky substituents) is at the same time the most stabilized by quasi-aromaticity (3.44|*e*|, *i.e.* ∼0.6|*e*| per a quasi-ring member, which is comparable to the corresponding value for pyrrole^60^). Interestingly, it is found for the first time, that the systematic increase of the Zn–N bond polarization when going from 1 to 3 reveals a strict correlation between EDM (*i.e.* indirectly the topology and the metal to BDA charge transfer) and π-electron delocalization: *R* = −0.986. In other words, the more twisted is the 7-MR, the more quasi-aromatic character is observed (Fig. 7). It demonstrates the two-folded role of bulky substituents: they are not only dispersion donors,^40–56^ but they also lead to amplification of the 7-MR twist (and enhanced quasi-aromaticity). Previously only the former feature has been recognized.^23–27^ Interestingly, the optimized BDA–Zn structure (without steric effects from the Ph units) has significantly reduced quasi-aromaticity compared to 1 and 2 (2.43|*e*|, *i.e.* ∼0.4|*e*| per a quasi-ring member, which is comparable to the corresponding value for furan),^60^ but at the same time, it is almost twice less aromatically-stabilized than its optimized BDA–Cd analogue (4.74|*e*|, *i.e.* ∼0.8|*e*| per a quasi-ring member, which is exactly between the corresponding values for pyrrole and benzene).^60^ Since both equilibrium structures have comparable average dihedral angles N–N–M–N, it is clearly the larger metal–nitrogen bond polarization (the charges *q*_Zn_ = +1.15, *q*_N_ = −0.9, EDM = 1.26 D in BDA–Zn compared to *q*_Zn_ = +0.72, *q*_N_ = −0.7, EDM = 0.68 D in BDA–Cd) that limits the π-electron delocalization in the 7-memberd quasi-aromatic unit (changes in the effectiveness of π-conjugation involving the 2pz orbitals of nitrogen atoms at close proximity of the metals are well marked in the EDDB_π_(r) isocontours, Fig. 7). Such inter-relation between the nature of metal–ligand bonding and quasi-aromaticity of the ligand has not been known before.^23–27^

## Conclusions

In summary, we successfully isolated and characterized the quasi-aromatic Möbius type zinc complexes [Zn(NCS)_2_L^I^] (1), [Zn_2_(μ_1,1_-N_3_)_2_(L^I^)_2_][ZnCl_3_(MeOH)]_2_·6MeOH (2) and [Zn(NCS)L^II^]_2_[Zn(NCS)_4_]·MeOH (3), fabricated from 1,2-diphenyl-1,2-bis((phenyl(pyridin-2 yl)methylene)hydrazono)ethane (L^I^) or benzilbis(acetylpyridin-2-yl)methylidenehydrazone (L^II^), respectively, and ZnCl_2_ mixed with NH_4_NCS or NaN_3_. The creation of 1–3 is dictated by both the bulkiness of the organic ligand and the nature of inorganic counter ion.

Complex 1 has a neutral discrete heteroleptic mononuclear structure with the Zn^II^ metal atom being chelated by one tetradentate ligand L^I^ and two N-bound NCS^−^ anions with the formation of a distorted trigonal-prismatic ZnN_6_ coordination core. The [Zn(NCS)_2_L^I^] monomers were found (due to the ETS-NOCV calculations) to be bonded to each other primarily through London dispersion forces exerted by the presence of bulky hydrophobic substituents. Contrary, complexes 2 and 3 exhibit a salt-like structure where the long-range electrostatic forces were found to be of prime importance additionally to more typical non-covalent interactions (O–H⋯O, C–H⋯Cl, C–H⋯S, C–H⋯π, π⋯π). In 2, the cation is a doubly charged centrosymmetric dinuclear structure with two Zn^II^ atoms linked *via* two μ_1,1_-N_3_^−^ anions. Each metal center is further linked by the tetracoordinate ligand L^I^. The anionic part has a discrete mononuclear composition [ZnCl_3_(MeOH)]^−^. Notably, the anionic part of 2 together with the lattice MeOH molecules produces a synthon of motif *R*^8^_8_(20) stabilized mostly by O–H⋯O and C–H⋯Cl interactions. Complex 3 is composed from two [Zn(NCS)L^II^]^+^ cations with the Zn^II^ atoms each being chelated by two pyridyl-imine fragments of L^II^ and further bound by one N-linked NCS^−^ anion. The anionic part of 3 is a doubly charged [Zn(NCS)_4_]^2−^ species. Long range electrostatic forces between [Zn(NCS)_4_]^2−^ and [Zn(NCS)L^II^]^+^ are responsible for the stability of 3 since pure π⋯π stacking between the [Zn(NCS)L^II^]^+^ units appeared to be repulsive. Finally, we have proven, by means of the EDDB^57–61^ study, that the seven-membered rings in 1–3 constitute a quasi-aromatic Möbius-type motif, though the absolute magnitude of such π-delocalization is notably weaker than in the corresponding cadmium-based analogs.^24,27^ Bulkiness of the ligands (L) are found not only to amplify London dispersion stabilization,^24,27,40–56^ but also influence the magnitude of quasi-π-delocalization (of Möbius-type) through modification of the polarity of Zn–L bonding.

## Experimental

### Materials

All chemicals and solvents were used from commercial sources without further purification. L^I^ and L^II^ were synthesized according to a literature method.^20^

### Physical measurements

FTIR spectra were recorded on a Bruker Tensor 27 FTIR spectrometer. Microanalyses were performed using a ElementarVario EL III analyzer.

### Synthesis

ZnCl_2_ (0.068 g, 0.5 mmol), NH_4_NCS (0.076 g, 1 mmol) or NaN_3_ (0.065 g, 1 mmol) and L^I^ or L^II^ (0.284 and 0.222 g, respectively; 0.5 mmol) were placed in the main arm of a branched tube. MeOH (15 mL) was carefully added to fill the arms. The tube was sealed and immersed in an oil bath at 60 °C while the branched arm was kept at ambient temperature. X-ray suitable crystals were formed during the next days in the cooler arm and were filtered off.

(1) Colorless block-like crystals. Yield: 0.248 g (66%). Anal. calc. for C_40_H_28_N_8_S_2_Zn (750.22) (%): C 60.04, H 3.76 and N 14.94; found: C 60.29, H 3.83 and N 14.77.

(2) Yellow block-like crystals. Yield: 0.156 g (64%). Anal. calc. for C_84_H_88_Cl_6_N_18_O_8_Zn_4_ (1951.98) (%): C 51.69, H 4.54 and N 12.92; found: C 51.56, H 4.61 and N 12.81.

(3) Yellow prism-like crystals. Yield: 0.174 g (73%). Anal. calc. for C_63_H_52_N_18_OS_6_Zn_3_ (1465.74) (%): C 51.62, H 3.58 and N 17.20; found: C 51.76, H 3.48 and N 17.33.

### ETS-NOCV charge and energy decomposition method

The Natural Orbitals for Chemical Valence (NOCV) *ψ*_i_ constitute the canonical representation for a differential density matrix Δ*P* (it is formed by subtracting the appropriate molecular fragments density matrices from a density matrix of a molecule under consideration) in which Δ*P* adopts a diagonal form. It gives rise to the corresponding eigenvalues *v*_i_ and the related vectors *ψ*_i_. NOCVs occur in pairs (*ψ*_−*k*_,*ψ*_*k*_) related to |*v*_*k*_| and they decompose overall deformation density Δ*ρ* into bonding components with different symmetries (Δ*ρ*_*k*_):
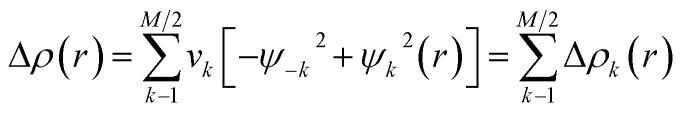


Usually, a few *k* allow to recover a major shape of Δ*ρ*. By combining NOCVs with ETS scheme in ETS-NOCV, one can obtain the related energetics, Δ*E*_orb_(*k*), in addition to qualitative picture emerging from Δ*ρ*_*k*_. ETS originally divides the total bonding energy, between fragments, Δ*E*_total_, into four distinct components: Δ*E*_total_ = Δ*E*_elstat_ + Δ*E*_Pauli_ + Δ*E*_orb_ + Δ*E*_dispersion_. The Δ*E*_elstat_ is an energy of quasi-classical electrostatic interaction between fragments. The next term, Δ*E*_Pauli_, is responsible for repulsive Pauli interaction between occupied orbitals on the two fragments. The third component, Δ*E*_orb_, is stabilizing and shows formation of a chemical bond (including polarizations). In the ETS-NOCV scheme Δ*E*_orb_ is expressed in terms of the eigenvalues *v*_*k*_ and diagonal Fock energy matrix elements *F*^TS^_i,i_ (transformed into NOCV representation) as:
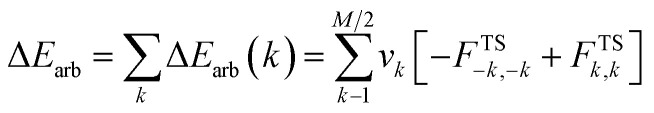


Finally, Δ*E*_dispersion_ denotes the semiempirical Grimme dispersion correction (D3).

### Single-crystal X-ray diffraction

The X-ray data were collected on a Bruker APEX-II CCD single crystal diffractometer using graphite-monochromated Mo-Kα radiation (*λ* = 0.71073 Å). The collected frames were integrated with the Saint^64^ software using a narrow-frame algorithm. Data were corrected for absorption effects using the multi-scan method in SADABS.^65^ The space groups were assigned using XPREP of the Bruker ShelXTL^66^ package, solved with ShelXT^66^ and refined with ShelXL^66^ and the graphical interface ShelXle.^67^ All non-hydrogen atoms were refined anisotropically. Hydrogen atoms attached to carbon were positioned geometrically and constrained to ride on their parent atoms. Figures were generated using the program Mercury.^68^

#### Crystal data for 1

C_40_H_28_N_8_S_2_Zn, *M*_r_ = 750.19 g mol^−1^, *T* = 296(2) K, monoclinic, space group *P*2_1_/*n*, *a* = 12.8146(10), *b* = 20.5355(17), *c* = 13.4451(11) Å, *β* = 98.225(1)°, *V* = 3501.7(5) Å^3^, *Z* = 4, *ρ* = 1.423 g cm^−3^, *μ*(Mo-Kα) = 0.863 mm^−1^, reflections: 5447 collected, 5447 unique, *R*_int_ = 0.034, *R*_1_(all) = 0.0363, w*R*_2_(all) = 0.0588, *S* = 1.026.

#### Crystal data for 2

C_76_H_56_N_18_Zn_2_, C_2_H_8_Cl_6_O_2_Zn_2_, 6(CH_4_O); *M*_r_ = 1951.9 g mol^−1^, *T* = 125(2) K, triclinic, space group *P*1̄, *a* = 11.836(4), *b* = 14.385(4), *c* = 14.807(4) Å, *α* = 68.045(3), *β* = 83.861(3), *γ* = 78.264(3)°, *V* = 2288.0(12) Å^3^, *Z* = 2, *ρ* = 1.417 g cm^−3^, *μ*(Mo-Kα) = 1.274 mm^−1^, reflections: 15 639 collected, 5424 unique, *R*_int_ = 0.053, *R*_1_(all) = 0.0648, w*R*_2_(all) = 0.1386, *S* = 1.092.

#### Crystal data for 3

2(C_29_H_24_N_7_SZn), C_4_N_4_S_4_Zn, CO; *M*_r_ = 1461.66 g mol^−1^, *T* = 100(2) K, triclinic, space group *P*1̄, *a* = 13.3235(9), *b* = 15.6906(10), *c* = 18.6880(13) Å, *α* = 65.795(2), *β* = 71.872(2), *γ* = 77.010(2)°, *V* = 3365.1(4) Å^3^, *Z* = 2, *ρ* = 1.443 g cm^−3^, *μ*(Mo-Kα) = 1.302 mm^−1^, reflections: 45 317 collected, 15 733 unique, *R*_int_ = 0.060, *R*_1_(all) = 0.1102, w*R*_2_(all) = 0.1712, *S* = 1.024.

## Contributions

Mariusz P. Mitoraj has planned and partially performed (ETS-NOCV) the theoretical calculations, written the manuscript text and analyzed the entire data. Farhad Akbari Afkhami has primarily done the experimental part. Ghodrat Mahmaoudi has planned the experimental research. Ali Akbar Khandar has supported the work, whereas Atash V. Gurbanov has synthesized the compounds. Fedor I. Zubkov has also participated in the synthesis of the compounds. Rory Waterman is a crystallographer of compound 1-2, whereas Himanshu Sekhar Jena is a crystallographer of system 3. D W. Szczepanik has done the aromaticity calculations. Damir A. Safin and Maria G. Babashkina have analysed and discussed the results as well as have written the manuscript.

## Conflicts of interest

There are no conflicts to declare.

## Supplementary Material

RA-009-C9RA05276C-s001

RA-009-C9RA05276C-s002
