# Correction: Structural versatility of the quasi-aromatic Möbius type zinc(ii)-pseudohalide complexes – experimental and theoretical investigations

**DOI:** 10.1039/c9ra90062d

**Published:** 2019-08-23

**Authors:** Mariusz P. Mitoraj, Farhad Akbari Afkhami, Ghodrat Mahmoudi, Ali Akbar Khandar, Atash V. Gurbanov, Fedor I. Zubkov, Rory Waterman, Maria G. Babashkina, Dariusz W. Szczepanik, Himanshu S. Jena, Damir A. Safin

**Affiliations:** Department of Theoretical Chemistry, Faculty of Chemistry, Jagiellonian University Gronostajowa 2 30-387 Cracow Poland mitoraj@chemia.uj.edu.pl; Department of Inorganic Chemistry, Faculty of Chemistry, University of Tabriz 51666-16471 Tabriz Iran; Department of Chemistry, Faculty of Science, University of Maragheh P.O. Box 55181-83111 Maragheh Iran mahmoudi_ghodrat@yahoo.co.uk; Department of Chemistry, Baku State University Z. Xalilov Str. 23 AZ1148 Baku Azerbaijan; Centro de Química Estrutural, Instituto Superior Técnico, Universidade de Lisboa Av. Rovisco Pais 1049-001 Lisboa Portugal; Organic Chemistry Department, Faculty of Science, Peoples’ Friendship University of Russia (RUDN University) 6 Miklukho-Maklaya St. Moscow 117198 Russian Federation; Department of Chemistry, University of Vermont 82 University Place Burlington VT 05405 USA; Institute of Condensed Matter and Nanosciences, Université Catholique de Louvain Place L. Pasteur 1 1348 Louvain-la-Neuve Belgium; COMOC, Department of Chemistry, Ghent University Krijgslaan 281-S3B Ghent-9000 Belgium; Innovation Center for Chemical and Pharmaceutical Technologies, Ural Federal University named after the First President of Russia B. N. Eltsin Mira Str. 19 Ekaterinburg 620002 Russian Federation damir.a.safin@gmail.com

## Abstract

Correction for ‘Structural versatility of the quasi-aromatic Möbius type zinc(ii)-pseudohalide complexes – experimental and theoretical investigations’ by Mariusz P. Mitoraj *et al.*, *RSC Adv.*, 2019, **9**, 23764–23773.

The authors regret that the affiliations of Maria G. Babashkinah and Damir A. Safin were incorrectly shown in the original manuscript. The corrected list of affiliations is as shown herein.

The Royal Society of Chemistry apologises for these errors and any consequent inconvenience to authors and readers.

## Supplementary Material

