# Supplementary material for: Structural versatility of the quasi-aromatic Möbius type zinc(ii)-pseudohalide complexes – experimental and theoretical investigations
Source: RSC Adv. 2019 Jul 31;9(41):23764–73. doi: 10.1039/c9ra05276c (PMC9069493; doi:10.1039/c9ra05276c)
Supplement: RA-009-C9RA05276C-s001 [file RA-009-C9RA05276C-s001.pdf]

Electronic Supplementary Material (ESI) for RSC Advances.
This journal is © The Royal Society of Chemistry 2019

Electronic Supplementary Information

Structural versatility of the *quasi*-aromatic Möbius type zinc(II)-pseudohalide complexes – experimental and theoretical investigations

Mariusz P. Mitoraj,*[a] Farhad Akbari Afkhami,[b] Ghodrat Mahmoudi,*[c] Ali Akbar Khandar,[b] Atash V. Gurbanov,[d,e] Fedor I. Zubkov,[f] Rory Waterman,[g] Maria G. Babashkina,[h] Dariusz W. Szczepanik[a], Himanshu S. Jena[i] and Damir A. Safin*[h]

[a]*Department of Theoretical Chemistry, Faculty of Chemistry, Jagiellonian University, Gronostajowa 2, 30-387 Cracow, Poland. E-mail: mitoraj@chemia.uj.edu.pl*

[b]*Department of Inorganic Chemistry, Faculty of Chemistry, University of Tabriz, 51666-16471, Tabriz, Iran*

[c]*Department of Chemistry, Faculty of Science, University of Maragheh, P.O. Box 55181-83111, Maragheh, Iran. E-mail: mahmoudi_ghodrat@yahoo.co.uk*

[d]*Department of Chemistry, Baku State University, Z. Xalilov Str. 23, AZ1148, Baku, Azerbaijan*

[e]*Centro de Química Estrutural, Instituto Superior Técnico, Universidade de Lisboa, Av. Rovisco Pais, 1049-001, Lisboa, Portugal*

[f]*Organic Chemistry Department, Faculty of Science, Peoples' Friendship University of Russia (RUDN University), 6 Miklukho-Maklaya St., Moscow, 117198, Russian Federation*

[g]*Department of Chemistry, University of Vermont, 82 University Place, Burlington, VT 05405, USA*

[h]*Institute of Chemistry, University of Tyumen, Perekopskaya Str. 15a, 625003 Tyumen, Russian Federation. E-mail: damir.a.safin@gmail.com, d.a.safin@utmn.ru*

[i]*COMOC, Department of Chemistry, Ghent University, Krijgslaan 281 - S3B, Ghent - 9000, Belgium*

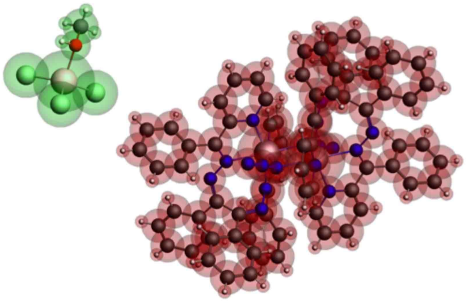

	$[ZnCl_3(MeOH)]^-/[Zn_2(\mu_{1,1}\text{-}N_3)_2(L^I)_2]^{2+}$
ΔE_{int}	–58.56
ΔE_{elstat}	–46.67
ΔE_{orb}	–11.77
$\Delta E_{dispersion}$	–0.27
ΔE_{Pauli}	0.06

$\Delta E_{int} = \Delta E_{elstat} + \Delta E_{orb} + \Delta E_{dispersion} + \Delta E_{Pauli}$

$\Delta\rho_{orb}$

$\Delta\rho < 0$ (outflow)

$\Delta\rho > 0$ (inflow)

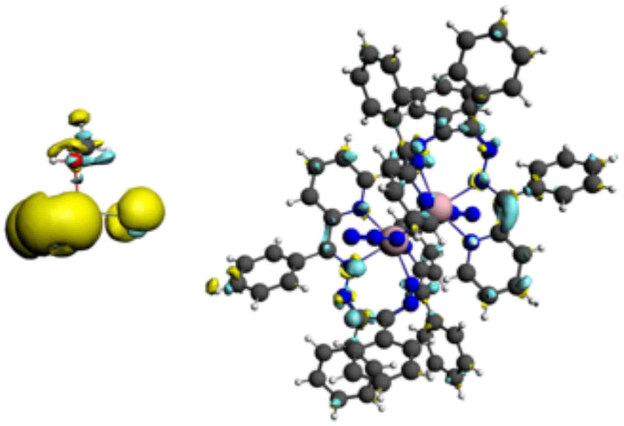

ΔE_{orb} = –11.77 kcal/mol

Fig. S1 (top) Results of the ETS-NOCV calculations describing interaction between **$[ZnCl_3(MeOH)]^-$** and **$[Zn_2(\mu_{1,1}\text{-}N_3)_2(L^I)_2]^{2+}$** in **2**. (bottom) The overall deformation density $\Delta\rho_{orb}$ with the corresponding orbital interaction energies ΔE_{orb}.

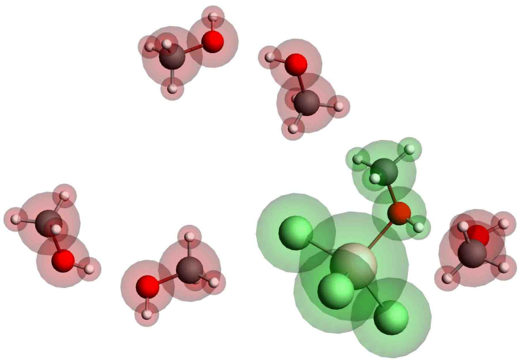

	O–H···O/C–H···Cl
ΔE_{int}	–13.17
ΔE_{elstat}	–16.30
ΔE_{orb}	–8.88
$\Delta E_{dispersion}$	–7.32
ΔE_{Pauli}	19.33

$\Delta E_{int} = \Delta E_{elstat} + \Delta E_{orb} + \Delta E_{dispersion} + \Delta E_{Pauli}$

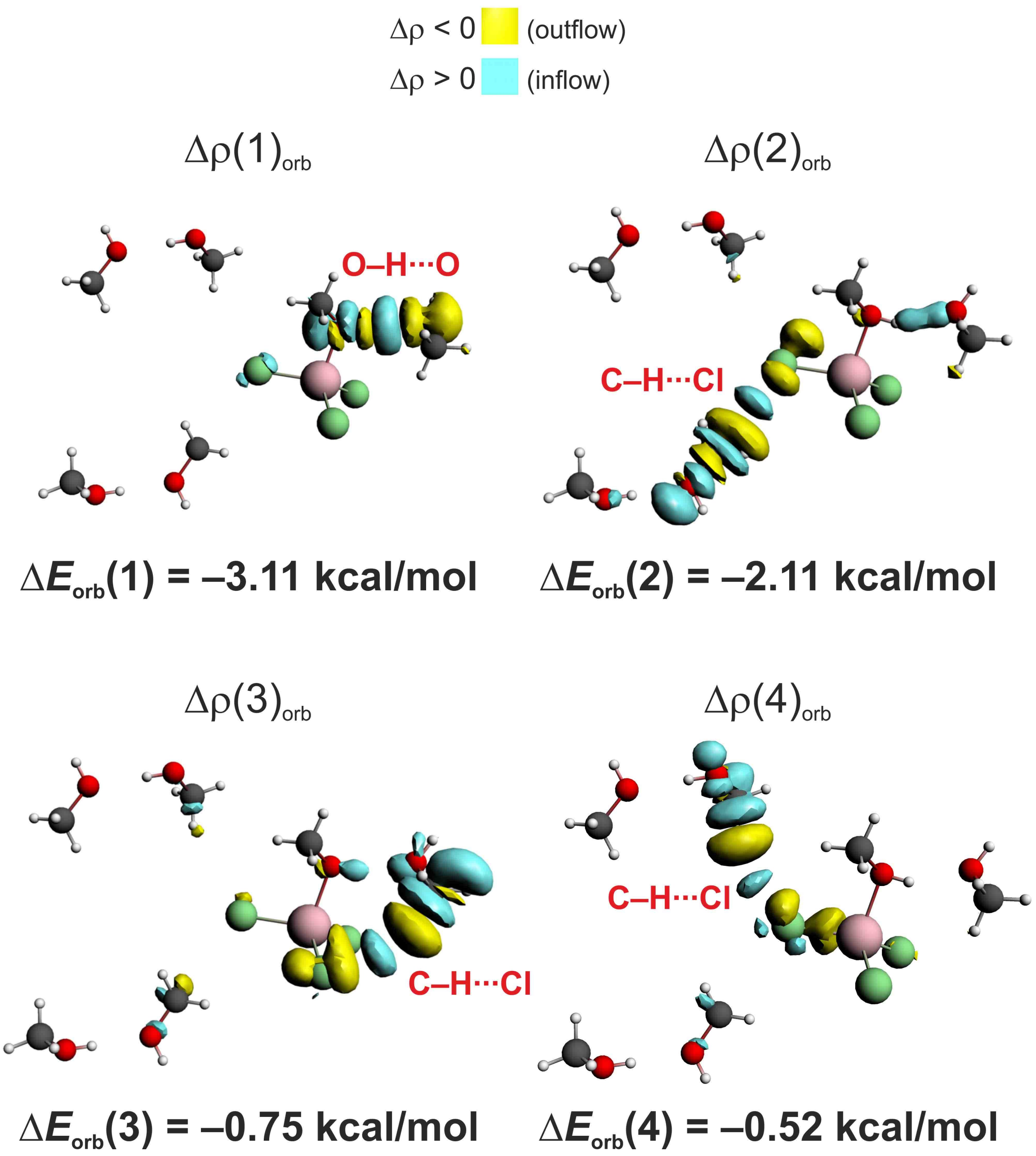

Fig. S2 (top) Results of the ETS-NOCV calculations describing interaction between **$[ZnCl_3(MeOH)]^–$** and methanol species in **2**. (bottom) The overall deformation density $\Delta\rho_{orb}$ and its NOCV contributions $\Delta\rho_{orb}(i)$ with the corresponding orbital interaction energies ΔE_{orb} and $\Delta E_{orb}(i)$.

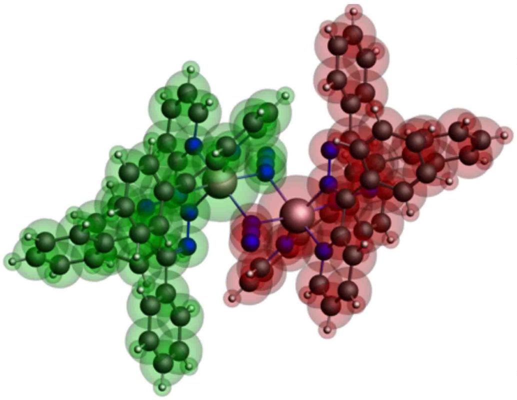

	Zn–N/C–H···π
ΔE_{int}	–31.63
ΔE_{elstat}	–63.33
ΔE_{orb}	–49.00
$\Delta E_{dispersion}$	–37.25
ΔE_{Pauli}	117.96

$\Delta E_{int} = \Delta E_{elstat} + \Delta E_{orb} + \Delta E_{dispersion} + \Delta E_{Pauli}$

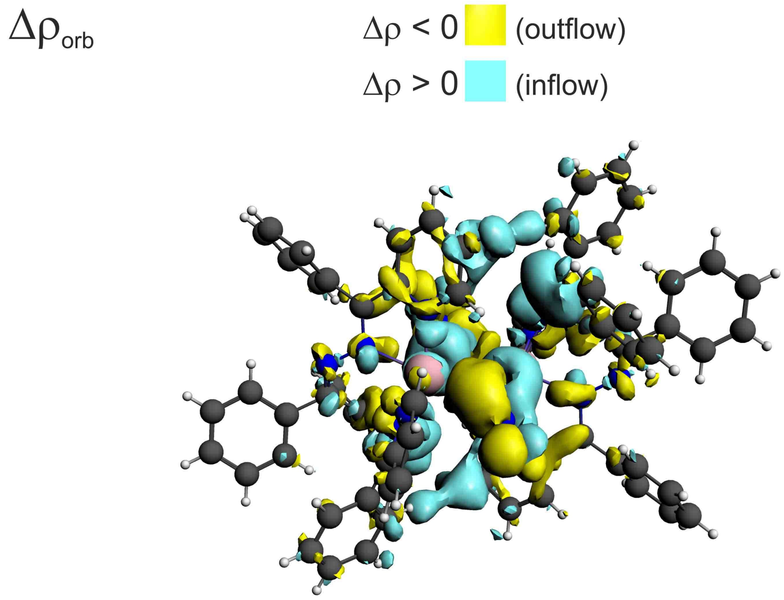

ΔE_{orb} = –49.00 kcal/mol

Fig. S3 (top) Results of the ETS-NOCV calculations describing Zn–N bonds in the $\mathbf{[Zn_2(\mu_{1,1}\text{-}N_3)_2(L^I)_2]^{2+}}$ synthon in **2**. (bottom) The overall deformation density $\Delta\rho_{orb}$ with the corresponding orbital interaction energies ΔE_{orb}.

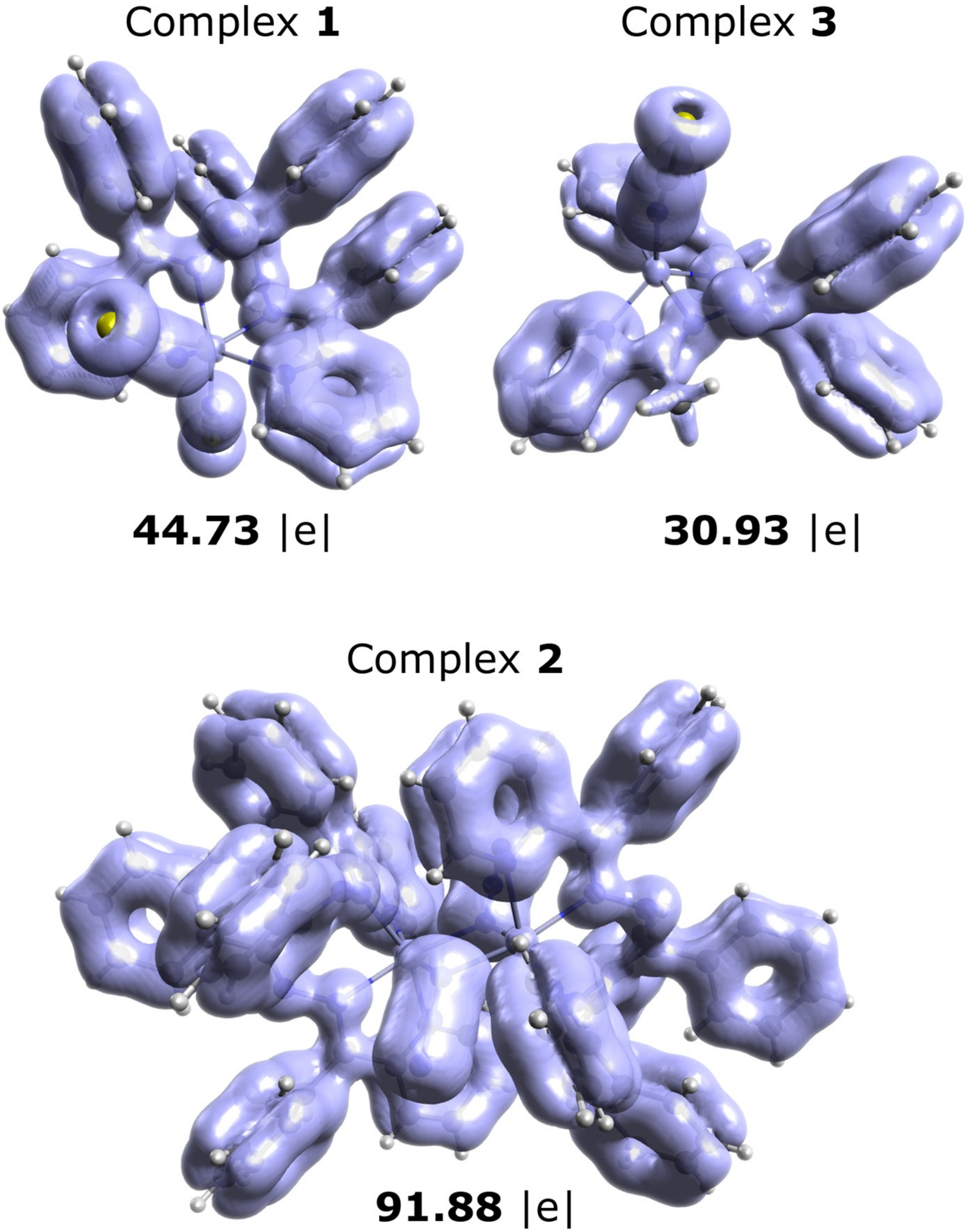

Fig. S4 The global EDDB isocontours and the corresponding electron populations of synthons from **1**–**3**.

Table S1. Coordination geometry around the Zn^{II} metal center in the structures of **1–3**, analyzed by the SHAPE 2.1 software

Complex	Pentagonal pyramid (C_{5v})	Octahedron (O_h)	Trigonal prism (D_{3h})	Pentagon (D_{5h})	Trigonal bipyramid (D_{3h})	Square pyramid (C_{4v})	Square (D_{4h})	Tetrahedron (T_d)	Seesaw (C_{2v})
**1**	14.387	7.891	**3.746**						
**2**	20.380	**2.701**	9.813				32.113	**0.610**	8.033
**3**				32.886, 32.446	**1.215**, **1.184**	4.168, 4.057	31.079	**0.157**	8.724

Table S2. Classic hydrogen bond lengths (Å) and angles (°) for **2**[a]

D–H···A	*d*(D–H)	*d*(H···A)	*d*(D···A)	∠(DHA)
O(1S)–H(1)···O(4S)[#1]	0.84	1.80	2.572(8)	153
O(2S)–H(2S)···O(3S)[#2]	0.84(8)	1.85(8)	2.689(10)	179(10)
O(3S)–H(3S)···Cl(1)[#3]	0.84(8)	2.49(10)	3.185(8)	141(15)
O(4S)–H(4S)···O(2S)[#4]	0.84	1.87	2.672(8)	159

[a]Symmetry transformations used to generate equivalent atoms: #1 *x*, *y*, *z*; #2 –*x*, 1 – *y*, 1 – *z*; #3 –1 + *x*, *y*, *z*; #4 1 + *x*, 1 + *y*, *z*.

Table S3. π···π interaction distances (Å) and angles (°) for **1–3**[a]

Complex	Cg(*I*)	Cg(*J*)	*d*[Cg(*I*)–Cg(*J*)]	α	β	γ	slippage
**1**[b]	Cg(4)	Cg(4)[#1]	3.5117(12)	0.02(10)	12.1	12.1	0.733
	Cg(6)	Cg(7)[#2]	3.7951(13)	9.25(11)	31.4	22.2	1.979
	Cg(7)	Cg(6)[#2]	3.7950(13)	9.25(11)	22.2	31.4	1.432
**2**[c]	Cg(6)	Cg(9)[#1]	3.962(4)	5.4(3)	27.3	24.8	1.818
	Cg(9)	Cg(6)[#1]	3.962(4)	5.4(3)	24.8	27.3	1.661
**3**[d]	Cg(3)	Cg(12)[#1]	4.088(3)	13.2(2)	37.7	24.8	2.502
	Cg(5)	Cg(9)[#2]	3.932(3)	11.9(2)	23.0	34.2	1.537
	Cg(9)	Cg(5)[#3]	3.932(3)	11.9(2)	34.2	23.0	2.210
	Cg(12)	Cg(3)[#4]	4.088(3)	13.2(2)	24.8	37.7	1.712

[a]Cg(*I*)–Cg(*J*): distance between ring centroids; α: dihedral angle between planes Cg(*I*) and Cg(*J*); β: angle Cg(*I*) → Cg(*J*) vector and normal to plane *I*; γ: angle Cg(*I*) → Cg(*J*) vector and normal to plane *J*; slippage: distance between Cg(*I*) and perpendicular projection of Cg(*J*) on ring *I*.

[b]Symmetry transformations used to generate equivalent atoms: #1 2 – *x*, –*y*, 2 – *z*; #2 *x*, *y*, *z*. Cg(4): N(6)–C(41)–C(42)–C(43)–C(44)–C(45), Cg(6): C(31)–C(32)–C(33)–C(34)–C(35)–C(36), Cg(7): C(111)–C(112)–C(113)–C(114)–C(115)–C(116).

[c]Symmetry transformations used to generate equivalent atoms: #1 1 – *x*, 1 – *y*, –*z*. Cg(6): C(21)–C(22)–C(23)–C(24)–C(25)–C(26), Cg(9): C(411)–C(412)–C(413)–C(414)–C(415)–C(416).

[d]Symmetry transformations used to generate equivalent atoms: #1 1 + *x*, *y*, *z*; #2 1 + *x*, 1 + *y*, *z*; #3 1 –1 + *x*, *y*, *z*; #4 1 –1 + *x*, –1 + *y*, *z*. Cg(3): N(1A)–C(8A)–C(9A)–C(19A)–C(7A)–C(28A), Cg(5): C(1A)–C(2A)–C(3A)–C(4A)–C(15A)–C(12A), Cg(9): N(2B)–C(6B)–C(15B)–C(13B)–C(11B)–C(14B); Cg(12): C(10B)–C(17B)–C(26B)–C(21B)–C(18B)–C(23B).

Table S4. C–H···π interaction distances (Å) and angles (°) for **1** and **2**[a]

Complex	C–H(*I*)	Cg(*J*)	*d*[H(*I*)–Cg(*J*)]	*d*[C–Cg(*J*)]	∠(CHCg)	γ
**1**[b]	C(25)–H(25A)	Cg(6)[#1]	2.77	3.609(2)	150	18.30
	C(44)–H(44A)	Cg(5)[#2]	2.87	3.690(2)	148	20.83
**2**[c]	C(36)–H(36A)	Cg(8)[#1]	2.86	3.569(7)	132	9.44

[a]Y(*I*)–Cg(*J*): distance of Y to ring centroid; X–Cg(*J*): distance of X to ring centroid; ∠(XYCg): angle X–Y–Cg; γ: angle Y(*I*) → Cg(*J*) vector and normal to plane *J*.
[b]Symmetry transformations used to generate equivalent atoms: #1 2 – *x*, –*y*, 1 – *z*; #2 2 – *x*, –*y*, 2 – *z*. Cg(5): C(171)–C(172)–C(173)–C(174)–C(175)–C(176).
[c]Symmetry transformations used to generate equivalent atoms: #1 *x*, *y*, *z*. Cg(8): C(111)–C(112)–C(113)–C(114)–C(115)–C(116).
